# Clinical outcomes for patients with thymoma and thymic carcinoma after undergoing different front‐line chemotherapy regimens

**DOI:** 10.1002/cam4.4711

**Published:** 2022-03-29

**Authors:** Wei‐Li Ma, Chia‐Chi Lin, Feng‐Ming Hsu, Jang‐Ming Lee, Jin‐Shing Chen, Yen‐Lin Huang, Yih‐Leong Chang, Chin‐Hao Chang, James Chih‐Hsin Yang

**Affiliations:** ^1^ Department of Oncology National Taiwan University Hospital Taipei Taiwan; ^2^ Graduate Institute of Oncology National Taiwan University College of Medicine Taipei Taiwan; ^3^ Graduate Institute of Clinical Medicine National Taiwan University College of Medicine Taipei Taiwan; ^4^ Division of Thoracic surgery, Department of Surgery National Taiwan University Hospital Taipei Taiwan; ^5^ Department of Surgical Oncology National Taiwan University Cancer Center Taipei Taiwan; ^6^ Department of Pathology National Taiwan University Cancer Center Taipei Taiwan; ^7^ Department of Medical Research National Taiwan University Hospital Taipei Taiwan; ^8^ Department of Medical Oncology National Taiwan University Cancer Center Taipei Taiwan

**Keywords:** front‐line chemotherapy, overall survival, thymic carcinoma, thymoma

## Abstract

**Background:**

Front‐line platinum‐base chemotherapy for advanced thymoma and thymic carcinoma (TC) improves resectability and prolongs patients' overall survival (OS). In this study, we evaluated patients' outcomes given different front‐line regimens: cisplatin, doxorubicin, and cyclophosphamide (CAP); cisplatin and etoposide (EP); or cisplatin and paclitaxel (TP).

**Materials and Methods:**

We retrospectively evaluated the medical records of patients with advanced thymoma and TC who were treated at our medical center between 2005 and 2015. We investigated objective response rates (ORRs), progression‐free survival (PFS), and OS after undergoing different front‐line regimens.

**Results:**

Among the 108 enrolled patients, 37 (34%) had thymoma and 71 (66%) had TC; 45 received CAP, 36 received EP, and 27 received TP regimens. The ORRs of patients receiving CAP, EP, and TP were 51%, 50%, and 41%, respectively. For patients with stage III and IVA disease, the median PFS after CAP, EP, and TP were 34.5, 26.4, and 18.0 months (*p* = 0.424), respectively, and the 5‐year OS rates were 84.9%, 70.6%, and 60.0% (*p* = 0.509). In patients with stage IVB disease, the median PFS were 9.4, 8.2, and 11.6 months after undergoing CAP, EP, and TP (*p* = 0.173), respectively, and the 5‐year OS rates were 41.1%, 39.1%, and 14.3% (*p* = 0.788). TC pathology subtype and liver metastasis were associated with poor OS. Three patients with stage IVB TC had an OS of more than 5 years.

**Conclusion:**

Different front‐line chemotherapy regimens may provide similar long‐term PFS and OS in patients with advanced thymoma and TC. In addition to TC and liver metastasis were associated with poor OS, other potential prognostic factors are warranted for studying.

## Graphical abstract

This study compared thymoma and thymic carcinoma patients’ objective response rates, progression‐free survival and overall survival after receiving three different front‐line chemotherapies: cisplatin, doxorubicin, and cyclophosphamide (CAP); cisplatin and etoposide (EP); and cisplatin and paclitaxel (TP). Patietns with stage III/IVA or stage IVB thymoma and thymic carcinoma had similar long‐term overall survival after receiving three different front‐line chemotherapy regimens. This paper also provided comprehensive clinical information and studied clinical prognostic factors in patients with advanced thymoma and thymic carcinoma.

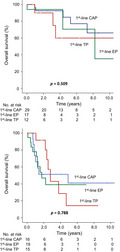

## 1 INTRODUCTION

Thymic cancer, which arises from thymic epithelium, includes thymoma, thymic carcinoma (TC), and other rare subtypes of malignancies.^1^ According to data from the Surveillance, Epidemiology, and End Results program, the annual incidence of thymoma in the United States is 0.13 per 100,000 population.^2^ Furthermore, the incidence of thymoma is higher in Asian–Pacific islanders and African Americans than in Caucasians.^3^ In Taiwan, the incidences of thymic cancer in men and women were 1.61 and 1.33 per 100,000 persons at risk, respectively, according to the 2016 Cancer Registry Annual Report.^4^

The primary treatment approach for thymic cancer without extra‐thoracic metastasis is complete resection.^5^, ^6^, ^7^, ^8^, ^9^ Notably, postoperative radiotherapy to tumor beds for patients at risk of microscopic residual disease or to gross tumors for patients with macroscopic incomplete resection was found to decrease recurrence rates, especially among those with stage III and IVA disease.^10^, ^11^, ^12^, ^13^ Platinum chemotherapy combined with anthracycline, etoposide, or paclitaxel are the most commonly used front‐line regimens for treating advanced thymic cancer. Importantly, these regimens improve the resectability of thymic tumors in the thoracic cavity and prolong patients' overall survival (OS).^14^, ^15^, ^16^

We had previously studied thymic cancer patients' outcomes according to different front‐line treatments, including up‐front surgery, induction chemotherapy followed by surgery, and no surgical treatment (i.e., medical treatment or with radiotherapy only). We found that up‐front surgery led to the best OS, and patients with advanced thymic cancer usually received induction chemotherapy with or without further surgery.^17^ Due to the rarity of thymic cancer, few studies have directly compared patients' outcomes according to different front‐line chemotherapies. With the exception of the pathological subtype of TC and advanced‐stage tumors without complete resection, adverse prognostic factors have rarely been discussed.^18^, ^19^

Therefore, this study was designed to compare objective response rates (ORRs), progression‐free survival (PFS), and OS in patients with thymoma and TC after undergoing treatment with different front‐line platinum‐based chemotherapy regimens. Moreover, we evaluated clinical prognostic factors for patients' long‐term OS.

## 2 METHODS

### 2.1 Clinicopathological features and treatment modalities

We retrospectively reviewed the medical records of patients with thymoma or TC who received front‐line chemotherapy regimens at our medical center from 2005 to 2015. The patients underwent pretreatment assessments, including a history report, physical examinations, complete blood cell counts, blood chemistry analysis, and whole‐body computed tomography (CT) for the purpose of staging. Positron emission tomography/CT was arranged as indicated for the specific condition. No patients received endobronchial ultrasound‐guided transbronchial needle aspiration for lymph‐node biopsy at initial staging, but chest surgeons arranged lymph‐node dissection during the operation if lymph node metastasis was suspected.

The enrolled patients were divided into three groups based on undergoing different front‐line chemotherapy regimens: group 1 comprised patients receiving a cisplatin, doxorubicin, and cyclophosphamide (CAP) regimen; group 2 comprised patients receiving a cisplatin and etoposide (EP) regimen; and group 3 comprised patients receiving a cisplatin and paclitaxel (TP) regimen. Attending physicians decided on one of these three front‐line chemotherapy regimens after discussing previous study results and treatment‐related side effects with their patients, and the final dosages were adjusted according to the patients' clinical conditions.^14^, ^15^, ^16^

The paclitaxel chemotherapy could be administered weekly or every 3 weeks at the attending physician's judgment. The attending physicians decided on the total number of chemotherapy cycles according to patients' tolerability and clinical benefit, and patients received the minimum of 2 cycles of chemotherapy. Since some patients presented with clinical stage III and IVA thymoma or TC, they needed to undergo surgery after receiving front‐line chemotherapy. These surgical procedures included total thymectomy and complete excision of gross tumors in the thoracic cavity. After surgery, patients received radiotherapy with 45–54 Gy for close (1–2 mm) or microscopically involved (<1 mm) resection margins, and with up to 60 Gy for gross residual tumors.^12^, ^13^

Pathological diagnosis of thymoma or TC was based on the 2015 World Health Organization (WHO) classification system, and two pathologists (Y.L.H. and Y.L.C.) with a professional focus on thoracic malignancies confirmed the diagnoses.^1^ Clinical staging was determined using the Masaoka–Koga staging system and the 8th edition of the TNM Classification of thymic epithelial malignant tumors.^20^, ^21^ The final visceral metastatic sites in all patients were reported during the last follow‐up visit.

### 2.2 Evaluation and ethics statements

Most patients underwent CT assessments every 3–6 months, according to the Taiwan National Health Insurance regulations. We administered a response evaluation to patients undergoing front‐line chemotherapy regimens according to Revised RECIST guidelines (version 1.1).^22^ PFS after front‐line chemotherapy was defined as the interval from the date of first‐line chemotherapy initiation to the date of any treatment failure, including disease recurrence, progression, or death. OS was defined as the interval from the date of diagnosis of thymoma or TC to the date of death from any cause.

Clinical stage III/IVA or stage IVB for thymic cancer strongly affects patients' prognoses and indications for treatment modalities. Most patients diagnosed with stage III/IVA thymic cancer receive curative‐intent treatment, while most stage IVB patients receive palliative‐intent treatments. Thus, our patients' long‐term PFS and OS in accordance with different front‐line chemotherapy regimens were analyzed according to stage III/IVA or stage IVB classifications.

The National Taiwan University Hospital Research Ethics Committee (NTUHREC) approved this retrospective study (NTUHREC No. 202106012RINA) and waived the informed consent, as all patient identifiers were removed from the dataset prior to data analysis. This study was conducted in accordance with the Declaration of Helsinki and its later amendments.

### 2.3 Statistical analysis

We compared the distributions of categorical variables for patients receiving different front‐line chemotherapy regimens using Fisher's exact test. Continuous variables and medians were compared using the Kruskal–Wallis test. Long‐term PFS and OS were calculated using the Kaplan–Meier method.

Univariate and multivariate Cox proportional hazard models were used to explore associations between OS and the following potential prognostic factors: patient age, sex, pathological subtypes, initial stages, front‐line chemotherapy regimens, and visceral metastasis. The *p*‐values of <0.05 were used to denote statistical significance.

## 3 RESULTS

### 3.1 Patient characteristics and treatment modalities

Table 1 shows the clinical characteristics of the 108 patients receiving front‐line chemotherapy. In total, 45 patients received CAP, 36 patients received EP, and 27 patients received TP chemotherapy regimens. The median age of the patients in the front‐line CAP regimen group was 57 years (range: 22–79 years), and 24 of these patients (53%) were male. Nine (20%) and 25 patients (55%) had WHO classifications of type B3 thymoma and TC, respectively. Sixteen patients (36%) had stage IVB disease, and 34 patients (76%) underwent surgery for thymic tumors. The pleura and pericardium were the most common metastatic sites (39 patients, 87%).

**TABLE 1:** Medical and demographic characteristics of patients with thymoma or thymic carcinoma receiving front‐line platinum‐based chemotherapy

	CAP regimen (*n* = 45)	EP regimen (*n* = 36)	TP regimen (*n* = 27)	*p* value
Age (years)				
Median (range)	57 (22–79)	51 (13–77)	58 (28–77)	0.191
>60, *n* (%)	18 (40)	12 (33)	13 (48)	0.493
Gender, *n* (%)				0.300
Male	24 (53)	23 (64)	12 (44)	
Female	21 (47)	13 (36)	15 (56)	
Aplastic anemia or Hb <10 g/dl at diagnosis, *n* (%)	2 (4)	4 (11)	0 (0)	0.156
Myasthenia gravis, *n* (%)	1 (2)	1 (3)	1 (4)	0.934
Pathology subtypes, *n* (%)				0.025^a^ ^,^ *
Thymoma				
A	2 (4)	1 (3)	0 (0)	
AB	2 (4)	2 (6)	1 (4)	
B1	2 (4)	2 (6)	0 (0)	
B2	5 (11)	6 (17)	0 (0)	
B3	9 (20)	2 (6)	3 (11)	
Thymic carcinoma				
Thymic carcinoma, NOS	15 (33)	16 (42)	13 (48)	
Squamous cell carcinoma	10 (22)	6 (17)	9 (33)	
Adenocarcinoma	0 (0)	1 (3)	1 (4)	
Stages according to the Masaoka‐koga system, *n* (%)				0.379
Stage III	13 (29)	8 (22)	7 (26)	
Stage IVA	16 (36)	9 (25)	5 (19)	
Stage IVB	16 (36)	19 (53)	15 (56)	
Surgery to resect thymus tumors, *n* (%)	34 (76)	15 (42)	16 (59)	0.008*
Metastatic sites, *n* (%)				
Pleural/pericardium	39 (87)	24 (67)	17 (63)	0.039*
Lung	31 (69)	32 (89)	17 (63)	0.039*
Liver	16 (36)	10 (28)	11 (41)	0.546
Bone	10 (22)	10 (28)	8 (30)	0.749
Brain	0 (0)	1 (3)	2 (7)	0.180

Abbreviations: CAP, cisplatin, doxorubicin, and cyclophosphamide; EP, cisplatin and etoposide; Hb, hemoglobin; TP, cisplatin and paclitaxel.

^a^ The pathology subtypes for Fisher's exact test were grouped by non‐B3 subtypes of thymoma, B3 subtype of thymoma, and thymic carcinoma.

* *p* < 0.05.

The median age of the 36 patients receiving front‐line EP chemotherapy was 51 years (range: 13–77 years), and 23 of these patients (64%) were male. Two patients (6%) had a WHO classification of type B3 thymoma, and 23 patients (62%) were diagnosed with TC. Nineteen patients (53%) had stage IVB disease. A total of 15 patients (42%) underwent surgery for thymic tumors, and 32 patients (89%) had lung metastases.

The median age of the patients in the front‐line TP chemotherapy group was 58 years (range: 28–77 years), and 12 of these patients (44%) were male. Twenty‐three (81%) patients had TC, and 15 (56%) had stage IVB disease. In total, 16 patients (59%) underwent surgery for thymic tumors, 17 patients (63%) had pleural and pericardial tumors, and 17 patients (63%) had lung metastases.

A total of 29 of 45 patients (64%) receiving a CAP regimen, 17 of 36 patients (47%) receiving an EP regimen, and 12 of 27 patients (44%) receiving TP chemotherapy were diagnosed with stage III/IVA cancer. These chemotherapy regimens were addressed in the neoadjuvant setting.

When comparing three groups of patients receiving different front‐line chemotherapy regimens, the vast majority of patients diagnosed with TC received the TP regimen (23 patients [85%]; *p* = 0.025). The vast majority of patients in the CAP regimen group underwent surgery (34 patients [76%]; *p* = 0.008). In addition, patients receiving front‐line CAP or EP regimens developed more pleural/pericardial or lung metastases, respectively (39 patients [87%] in the CAP group; *p* = 0.039; 32 patients [89%] in the EP group; *p* = 0.039). Among a total of 37 (out of 108) patients who developed liver metastases, six and 31 patients were diagnosed with thymoma and TC, respectively. All 37 patients had metastases in the liver parenchyma and two patients also had liver surface involvement due to thymic tumors seeding. Table S1 shows the number of patients in each front‐line chemotherapy group according to TNM classification staging for thymic epithelial malignant tumors.

### 3.2 Clinical outcomes for patients undergoing different treatment modalities

Table 2 shows the treatment responses of patients receiving different front‐line chemotherapy regimens. In total, 23, 18, and 11 patients in the CAP, EP, and TP chemotherapy groups had partial responses to front‐line chemotherapy, and the ORRs associated with these regimens were 51%, 50%, and 41%, respectively. In patients with thymoma, the ORRs to CAP, EP, and TP regimens were 65%, 62%, and 50%, respectively. In patients with TC, the ORRs associated with CAP, EP, and EP regimens were 40%, 43%, and 39%, respectively. The ORRs were generally higher in thymoma than in TC. In total, 52 patients had a partial response to front‐line chemotherapy regimens, and 40 patients (77%) received additional surgery or radiotherapy to address thymic tumors. Thirteen patients (25%) continued the same chemotherapy regimens after undergoing surgery or radiotherapy.

**TABLE 2:** Clinical response of patients with thymoma or thymic carcinoma after undergoing front‐line platinum‐based chemotherapy

	CAP regimen (***n*** = 45)		EP regimen (***n*** = 36)		TP regimen (***n*** = 27)	
Response (%)						
CR	0 (0)		0 (0)		0 (0)	
PR	23 (51)		18 (50)		11 (41)	
SD	14 (31)		12 (33)		11 (41)	
PD	8 (18)		6 (17)		5 (19)	
	Thymoma (***n*** = 20)	Thymic carcinoma (***n*** = 25)	Thymoma (***n*** = 13)	Thymic carcinoma (***n*** = 23)	Thymoma (***n*** = 4)	Thymic carcinoma (***n*** = 23)
Response (%)						
CR	0 (0)	0 (0)	0 (0)	0 (0)	0 (0)	0 (0)
PR	13 (65)	10 (40)	8 (62)	10 (43)	2 (50)	9 (39)
SD	6 (30)	8 (32)	4 (31)	8 (35)	2 (50)	9 (39)
PD	1 (5)	7 (28)	1 (8)	5 (22)	0 (0)	5 (22)

Abbreviations: CAP, cisplatin, doxorubicin, and cyclophosphamide; CR, complete response; EP, cisplatin and etoposide; PD, progressive disease; PR, partial response; SD, stable disease; TP, cisplatin and paclitaxel.

**FIGURE 1:**
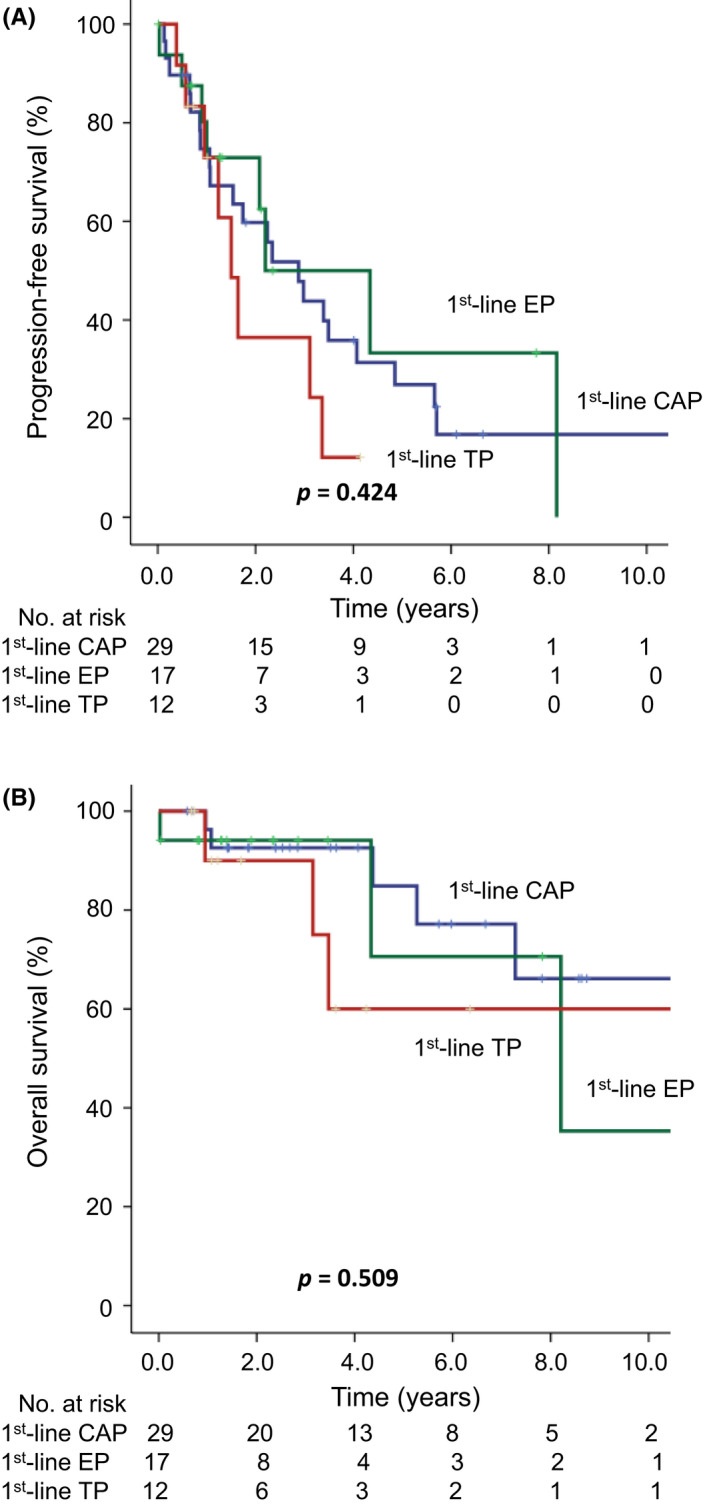
Progression‐free survival (PFS) and overall survival (OS) for stage III/IVA patients with thymoma or thymic carcinoma receiving front‐line cisplatin, doxorubicin, and cyclophosphamide (CAP), cisplatin and etoposide (EP), or cisplatin and paclitaxel (TP) chemotherapy regimens. (A) The median PFS durations for the CAP, EP, and TP chemotherapy regimens were 34.5, 26.4, and 18.0 months, respectively (*p* = 0.424). (B) The 5‐year OS rates were 84.9%, 70.6%, and 60.0%, respectively (*p* = 0.509)

**FIGURE 2:**
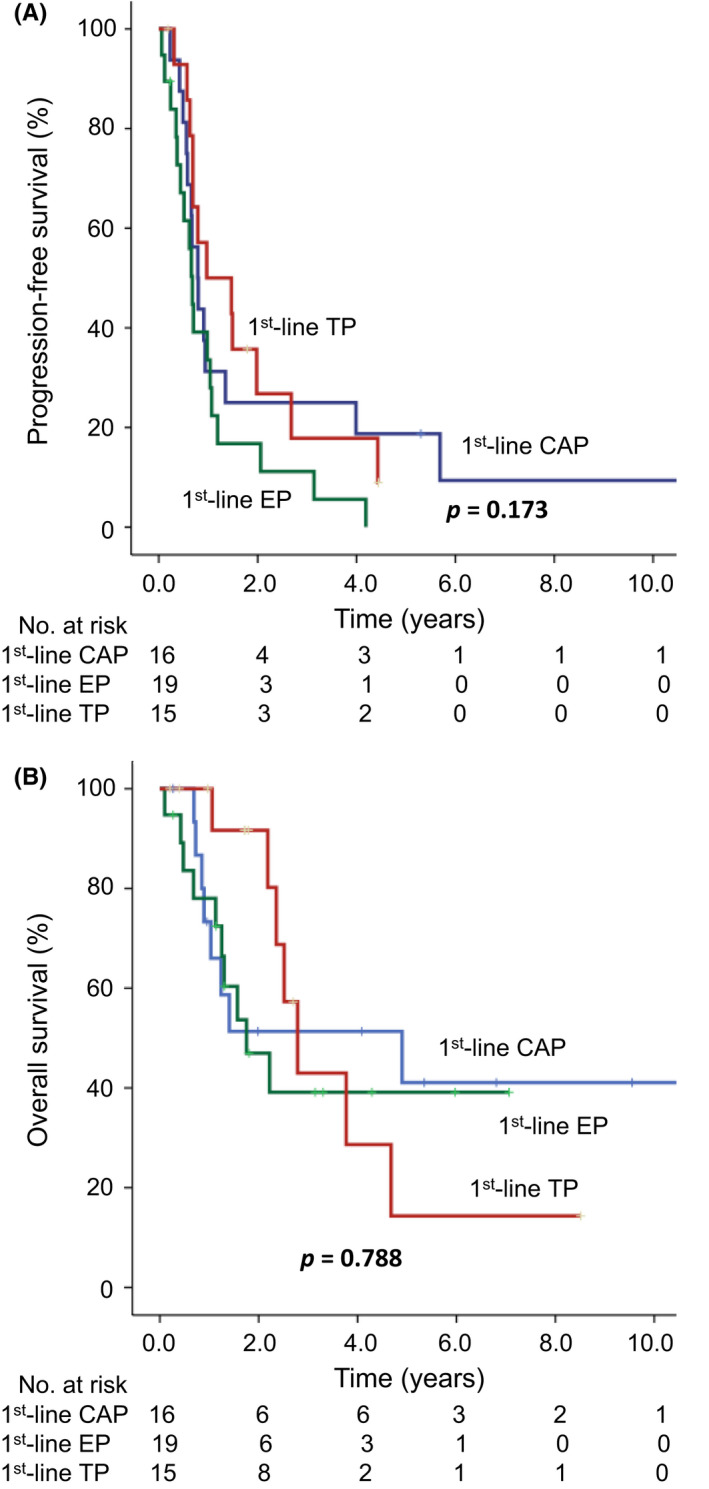
Progression‐free survival (PFS) and overall survival (OS) for patients with stage IVB thymoma or thymic carcinoma receiving front‐line cisplatin, doxorubicin, and cyclophosphamide (CAP), cisplatin and etoposide (EP), or cisplatin and paclitaxel (TP) chemotherapy regimens. (A) The median PFS durations for the CAP, EP, and TP chemotherapy regimens were 9.4, 8.2, and 11.6 months, respectively (*p* = 0.173). (B) The 5‐year OS rates were 41.1%, 39.1%, and 14.3%, respectively (*p* = 0.788)

Among the 58 patients with stage III or IVA thymoma and TC, 29 patients received front‐line CAP, 17 received front‐line EP, and 12 patients received front‐line TP regimens. Forty‐four patients (76%) received surgery and 58 patients (100%) received radiotherapy for thymic malignancies after undergoing chemotherapy. Thus, a total of 58 patients with stage III and IVA cancer received curative‐intent treatments. Patients' median PFS were 34.5, 26.4, and 18.0 months (*p* = 0.424) (Figure 1A), and their 5‐year OS rates were 84.9%, 70.6%, and 60.0%, respectively (*p* = 0.509) (Figure 1B). In patients with stage IVB disease, 16, 19, and 15 patients received front‐line CAP, EP, and TP regimens, respectively. In a total of 50 stage IVB patients, 21 patients (42%) received surgery and 31 (62%) patients received radiotherapy for thymic malignancies after undergoing chemotherapy. All 50 stage IVB patients received palliative‐intent treatments. Patients' median PFS were 9.4, 8.2, and 11.6 months (*p* = 0.173) (Figure 2A), and their 5‐year OS rates were 41.1%, 39.1%, and 14.3%, respectively (*p* = 0.788) (Figure 2B).

The PFS or OS of patients with stage III/IVA or stage IVB thymoma or of TC patients according to different front‐line chemotherapy regimens are listed on Table S2. In stage III/IVA thymoma or TC, the median PFS after receiving different front‐line chemotherapy regimens ranged from 18.0 to 35.8 months and from 10.4 to 26.4 months, respectively. In stage IVB TC, the median PFS after undergoing different front‐line chemotherapy regimens ranged from 7.5 to 9.5 months. Thus, chemotherapy led to a longer PFS in thymoma patients than in TC patients, and the differences in PFS between different front‐line chemotherapy regimens were statistically insignificant.

Table S3 shows the 51 patients with thymoma or TC received second‐line systemic treatments, including doxorubicin, etoposide, taxane, gemcitabine, and fluorouracil‐based regimens, oral cyclophosphamide, or pembrolizumab. Oral cyclophosphamide, fluorouracil or etoposide‐based regimens were the most commonly used regimens, accounting for 12, 11, and 10 of patients, respectively. Overall, four of 13 patients (31%) with thymoma and seven of 38 patients (18%) with TC had a partial response to second‐line systemic treatment regimens.

### 3.3 Treatment‐related side effects

The most common side effects in patients receiving a CAP regimen were leukopenia, which occurred in 34 (76%) patients, including 22 (49%) patients with grade I/II and 12 (27%) patients with grade III/IV leukopenia. In addition, grade I/II thrombocytopenia and anemia occurred in 11 (24%) and eight (18%) patients, respectively. In patients receiving an EP regimen, 15 (42%) patients had grade I/II leukopenia and nine (25%) patients had grade III/IV leukopenia, respectively. Grade I/II thrombocytopenia and anemia occurred in eight (22%) and seven (19%) patients, respectively. Moreover, 10 (37%) patients had grade I/II leukopenia and five (19%) patients had grade III/IV leukopenia after undergoing a TP regimen. Grade I/II thrombocytopenia, anemia, and neuropathy occurred in four (15%), five (19%), and 17 (63%) patients, respectively.

### 3.4 Univariate and multivariate analyses of prognostic factors for OS

Table 3 shows the results of univariate and multivariate analyses evaluating prognostic factors for OS. Univariate analyses evaluating OS assessed the prognostic impacts of patient age, sex, pathological subtype, initial staging, and metastatic sites after undergoing different front‐line chemotherapy regimens. Based on the univariate analysis, the different evaluated front‐line chemotherapy regimens were not associated with a statistically significant prognostic impact with regard to OS. The pathological subtype of TC (hazard ratio [HR], 17.46; confidence interval [Cl], 2.35–129.61; *p* = 0.005), Masaoka–Koga system stage IVB disease (HR, 3.67; Cl, 1.39–9.65; *p* = 0.009), and liver metastasis (HR, 2.77; Cl, 1.39–5.54; *p* = 0.004) were associated with poor survival. In contrast, pleural or pericardial metastasis (HR, 0.37; Cl, 0.18–0.76; *p* = 0.007) was associated with a favorable prognosis. In multivariate analysis, pathological subtype of TC (HR, 14.65; Cl, 1.93–111.24; *p* = 0.009) and liver metastasis (HR, 2.10; Cl, 1.04–4.25; *p* = 0.040) were associated with poor survival.

**TABLE 3:** Univariate and multivariate analysis of prognostic factors for overall survival in advanced thymoma and thymic carcinoma

Variables	Hazard ratio (95% Cl)	*p* value
Univariate analysis		
Front‐line chemotherapy regimens		
CAP	Reference	
EP	1.88 (0.86–4.08)	0.112
TP	1.71 (0.74–3.92)	0.209
Age > 60 years	1.48 (0.77–2.86)	0.241
Gender (male vs. female)	1.73 (0.88–3.38)	0.112
Pathology		
Thymoma (non‐B3 subtypes)	Reference	
Thymoma (B3 subtype)	3.05 (0.28–33.67)	0.363
Thymic carcinoma	17.46 (2.35–129.61)	0.005*
Stages (Masaoka‐Koga system)		
III	Reference	
IVA	1.04 (0.32–3.40)	0.955
IVB	3.67 (1.39–9.65)	0.009*
Metastatic sites		
Pleura/pericardium	0.37 (0.18–0.76)	0.007*
Lung	1.28 (0.58–2.82)	0.534
Liver	2.77 (1.39–5.54)	0.004*
Bone	1.63 (0.83–3.23)	0.159
Multivariate analysis		
Pathology		
Thymoma (non‐B3 subtypes)	Reference	
Thymoma (B3 subtype)	3.08 (0.27–34.68)	0.363
Thymic carcinoma	14.65 (1.93–111.24)	0.009*
Metastatic sites		
Pleura/pericardium	0.49 (0.23–1.05)	0.065
Lung	1.22 (0.54–2.76)	0.636
Liver	2.10 (1.04–4.25)	0.040*
Bone	1.05 (0.51–2.16)	0.885

Abbreviations: CAP, cisplatin, doxorubicin, and cyclophosphamide; CI, confidence interval; EP, cisplatin and etoposide; TP, cisplatin and paclitaxel.

* *p* < 0.05.

Table S4 lists the three patients with stage IVB TC who had a survival time of longer than 5 years. These patients initially had metastases in the pleural pericardium, lung, lymph nodes, and/or bone, but did not have liver metastasis after systemic treatment. The treatment responses to the front‐line chemotherapy regimens were either partial responses or stable disease.

## 4 DISCUSSION

Although platinum combined with anthracycline, etoposide, or taxane are among the most common front‐line chemotherapy regimens for treating thymoma and TC, studies comparing patients' long‐term outcomes are limited. One study conducted by Fornasiero et al. assessed 32 patients with stage III or IV thymoma who received the first‐line cisplatin, doxorubicin, vincristine, and cyclophosphamide (ADOC) regimen. They found an ORR to the ADOC regimen of 91.8%, and likewise found that the median OS for patients undergoing this regimen was 15 months.^14^ Another study by Loehrer et al., which included 29 patients with thymoma and one patient with TC in metastatic or recurrent disease who received first‐line CAP regimen, reported an ORR of 50%. Moreover, these researchers found that the time to treatment failure and the median OS time were 18.4 and 37.7 months, respectively.^23^ Kim et al. conducted a phase II study using a multidisciplinary approach of induction chemotherapy followed by surgical resection, radiation therapy, and consolidation chemotherapy for patients with unresectable malignant thymoma. Twenty‐two patients received the CAP regimen with prednisone. The ORR was 77% after induction chemotherapy, and the 5‐year PFS and OS rates were 77% and 95%, respectively.^24^

In a study by Giaccone et al., 16 patients with recurrent or metastatic malignant thymoma received EP chemotherapy, yielding an ORR of 56% and median PFS and OS durations of 2.2 and 4.3 years, respectively.^15^ The combination of etoposide, ifosfamide, and cisplatin treatment was also studied in 28 patients with thymoma or TC. This regimen produced an ORR of 32% and a median OS of 31.6 months.^25^ When taxane was combined with platinum chemotherapy as a novel front‐line treatment regimen, carboplatin instead of cisplatin became the most studied treatment. For example, in a study by Lemma et al., patients with unresectable thymoma (*n* = 21) or TC (*n* = 23) were treated with chemotherapy regimens containing carboplatin and paclitaxel. These researchers found ORRs of 42.9% and 21.7% in patients with thymoma or TC, respectively. The median PFS durations in the thymoma and TC cohorts was 16.7 and 5.0 months, respectively. Moreover, the median OS durations in the thymoma and TC cohorts were “not reached” and 20.0 months, respectively.^16^ Additionally, a study by Hirai et al. that included 40 patients with TC who were treated with carboplatin and paclitaxel found an ORR of 36% and a PFS of 7.5 months.^26^

In this study, we divided patients into three groups according to different front‐line chemotherapy regimens. Notably, more than 80% of the patients in the TP group were diagnosed with TC. The percentages of patients with stage IVB disease in the CAP, EP, and TP groups were approximately 30%, 50%, and 50%, respectively. Moreover, the ORRs of patients with thymoma or TC receiving front‐line chemotherapy were nearly 60% or 40%, respectively. Importantly, the ORRs observed in each chemotherapy group in this study were not consistent with those reported in previous studies. This may be due to the evaluation of treatment response by different attending physicians in our study as well as differences in imaging follow‐up intervals. However, in the current study, patients with TC were less responsive to platinum‐based chemotherapy regimens than were those with thymoma, which conforms to the findings of previous investigations. Moreover, more patients in the front‐line TP chemotherapy group had stage IVB disease and TC. This explains why patients in this group had a worse ORR to chemotherapy as well as a worse OS as compared with those receiving front‐line CAP or EP chemotherapy regimens. Considering that the OS differences between the front‐line chemotherapy groups were not statistically significant within either the Kaplan–Meier curve or the univariate analysis, we speculated that front‐line CAP, EP, and TP regimens produce similar results in terms of long‐term OS (Table 4).

**TABLE 4:** Comparing clinical outcomes after undergoing different front‐line chemotherapy regimens in patients with thymoma or thymic carcinoma

Studies and first‐line regimens	Patient no.	Stages	Histology subtypes	ORR (CR rate)	Survival
Platinum combined with anthracycline					
Fornasiero et al.^14^ (ADOC regimen)	32	Stage III or IV	Thymoma	ORR 91.8% (CR 43%)	Median OS: 15 mos.
Loehrer et al.^23^ (CAP regimen)	30	Recurrent or metastatic	Thymoma: 29 TC: 1	ORR 50% (CR 10%)	Median time to treatment failure: 18.4 mos. Median OS: 37.7 mos
Kim et al.^24^ (CAP with prednisone regimen)	22	Stage III: 11 Stage IVA: 10 Stage IVB: 1	Thymoma	ORR 77% (CR 14%)	5‐year PFS rate: 77% 5‐year OS rate: 95%
Platinum combined with etoposide					
Giaccone et al.^15^ (EP regimen)	16	Locally advanced or metastatic	Thymoma	ORR 56% (CR 31%)	Median PFS: 2.2 yrs. Median OS: 4.3 yrs
Loehrer et al.^25^ (VIP regimen)	28	Stage III: 6 Stage IVA: 13 Stage IVB: 9	Thymoma: 20 TC: 8	ORR 32% (CR 0%)	Median duration of response: 11.9 mos Median OS: 31.6 mos
Platinum combined with taxane					
Lemma et al.^16^ (TCar regimen)	44	Stage III: 7 Stage IVA: 22 Stage IVB: 15	Thymoma: 21 TC: 23	Thymoma: 42.9% (CR 14.2%) TC: 21.7% (CR 0%)	Thymoma Median PFS: 16.7 mos Median OS: not reached TC Median PFS: 5.0 mos Median OS: 20.0 mos
Hirai et al.^26^ (TCar regimen)	39	Stage III: 3 Stage IVA: 10 Stage IVB: 26	TC	ORR 36% (CR 3%)	Median PFS: 7.5 mos Median OS: not reached
Present study	108	Stage III: 28 Stage IVA: 30 Stage IVB: 50	Thymoma: 37 TC: 71	CAP ORR 51% (CR 0%) EP ORR 50% (CR 0%) TP ORR 41% (CR 0%)	5‐year OS, stage III/IVA CAP (84.9%) vs. EP (70.6%) vs. TP (60.0%) 5‐year OS, stage IVB CAP (41.1%) vs. EP (39.1%) vs. TP (14.3%)

Abbreviations: ADOC, cisplatin, doxorubicin, vincristine, and cyclophosphamide; CAP, cisplatin, doxorubicin, and cyclophosphamide; CR, complete response; EP, cisplatin and etoposide; mos, months; ORR, objective response rate; OS, overall survival; PFS, progression‐free survival; TC, thymic carcinoma; TCar, carboplatin and paclitaxel; TP, cisplatin and paclitaxel; VIP, etoposide, ifosfamide, and cisplatin; yrs, years.

With regard to evaluating second‐line systemic therapy, the patients in this study received doxorubicin, etoposide, or taxanes as they did not receive these drugs in first‐line chemotherapy. Chemotherapy with gemcitabine or fluorouracil and pembrolizumab, a monoclonal antibody targeting programmed death ligand 1 (PD‐1), has been studied in thymic cancer.^27^, ^28^, ^29^, ^30^, ^31^ Notably, the patients enrolled in this study mostly received oral cyclophosphamide‐, fluorouracil‐, or etoposide‐based regimens as second‐line treatments. The ORRs in thymoma or TC patients who received second‐line treatments were 18% and 31%, respectively. Importantly, a study by Merveilleux du Vignaux et al. found that TP, EP, and CAP regimens were the most commonly used second‐line systemic treatments and that the ORRs in patients with thymoma and TC undergoing second‐line treatments were 39% and 19%, respectively.^32^ Although the distributions of second‐line systemic treatments in this report were different from those in the latter study, our patients demonstrated similar ORRs with regard to second‐line systemic treatments.

In Taiwan, attending physicians may choose different chemotherapy regimens to treat patients with thymic cancer according to the results of previous studies, medical guidelines, and clinical judgment. With regard to first‐line platinum‐based chemotherapy regimens, taxane is not covered by national health insurance and patients must, therefore, pay out‐of‐pocket expenses. This may have caused fewer of our patients to select front‐line TP (27 patients) as compared with EP (36 patients) and CAP (45 patients) regimens. In addition, with regard to later‐line treatments, patients still need to pay out‐of‐pocket costs for gemcitabine, taxane, pembrolizumab, or oral fluorouracil‐based regimens. In contrast, infused fluorouracil is covered by national health insurance. Although capecitabine or S‐1 were the most studied fluorouracil‐based regimens in later‐line treatments, more than half of the patients received infused fluorouracil‐based treatment in our study. In addition, some patients received oral cyclophosphamide in later‐line treatments. Metronomic oral cyclophosphamide may exert anti‐tumor effect by immunomodulation, though this treatment strategy was not well studied in thymic malignancies.^33^

Prior studies have reported that advanced Masaoka–Koga stage and incomplete resection are the two leading prognostic factors for poor OS in patients with thymoma and TC.^34^, ^35^ Compared with other WHO histological thymoma subtypes, the B3 type often presents at later stages and is associated with unfavorable outcomes.^36^, ^37^ In fact, some patients with thymoma present with pure red cell aplasia, which is associated with poor prognoses.^38^ Moreover, Okuma et al. examined multiple prognostic factors in patients with recurrent or metastatic thymic carcinoma and concluded that bone metastasis, liver metastasis, and hypoalbuminemia were poor prognostic factors based on the results of a multivariate analysis.^39^ In this study, patients with stage IVB disease, the pathological subtype of TC, and liver metastasis showed poor OS on univariate analysis. Although patients with B3 thymoma had a higher HR with regard to poor OS than those with non‐B3 thymoma on univariate analysis, the low number of patients in the B3 thymoma group likely caused this difference to be statistically insignificant. Moreover, patients with pleural or pericardial metastasis showed a favorable OS on univariate analysis, possibly because these metastases are mostly confirmed in locally advanced disease after surgery. In the multivariate analysis, we confirmed that the pathological subtype of TC and liver metastasis were each associated with poor OS. In contrast, our three patients with stage IVB TC had a long‐term OS of longer than 5 years, thus highlighting the need to study other prognostic factors within future research.

The limitations of this study include its retrospective design and the heterogeneous patient sample comprising different thymic neoplasm subtypes, diverse treatment protocols, and varying imaging follow‐up intervals. The study data were recorded from 2005 to 2015, and therefore, the use of the latest surgery and clinical care techniques might have resulted in improved outcomes. Moreover, the number of enrolled patients was low, especially after grouping patients according to different front‐line chemotherapy regimens. Nevertheless, this study presented the substantial strength of evaluating clinical characteristics, long‐term outcomes, and prognostic factors for patients receiving different front‐line chemotherapy regimens. Additional studies are warranted to investigate other potential molecular prognostic factors of thymic neoplasms.

## 5 CONCLUSIONS

In this retrospective study conducted at a single medical center, we identified 108 patients with thymoma or TC who had undergone front‐line chemotherapy within an 11‐year interval. We found that long‐term PFS and OS among patients with stage III/IVA and stage IVB diseases were similar after undergoing different front‐line chemotherapy regimens. However, in the multivariate analysis, patients with TC or liver metastasis showed poor OS. Rarely, patients with stage IVB TC achieved a long‐term OS. Additional studies investigating other molecular prognostic factors are warranted.

## CONFLICT OF INTEREST

The authors have no conflicts of interest to declare.

## AUTHOR CONTRIBUTIONS

Wei‐Li Ma and Chia‐Chi Lin were responsible for literature collection, data management and interpretation, and manuscript writing. Wei‐Li Ma, Chia‐Chi Lin, Feng‐Ming Hsu, Jang‐Ming Lee, Jin‐Shing Chen, Yen‐Lin Huang, and Yih‐Leong Chang contributed to patient care and clinical data. Wei‐Li Ma and Chin‐Hao Chang were responsible for data management and interpretation and statistical analysis. Wei‐Li Ma and James Chih‐Hsin Yang treated patients; planned, designed, and coordinated the study throughout the study period; and wrote the manuscript.

## ETHICS APPROVAL

The National Taiwan University Hospital Research Ethics Committee (NTUHREC) approved this retrospective study (NTUHREC No. 202106012RINA) and waived the requirement for informed consent. The medical charts were retrospectively reviewed and all processes was carried out in accordance with the principles of the Declaration of Helsinki.

## Supporting information


Table S1–S4

## Data Availability

The datasets used and analyzed within the current study are available from the corresponding author on reasonable request.
