# Supplementary material for: Clinical outcomes for patients with thymoma and thymic carcinoma after undergoing different front‐line chemotherapy regimens
Source: Cancer Med. 2022 Mar 29;11(18):3445–56. doi: 10.1002/cam4.4711 (PMC9487882; doi:10.1002/cam4.4711)
Supplement: Supplementary file 1 — Table S1–S4 [file CAM4-11-3445-s001.docx]

**Supplementary Material**

| **Supplementary Table 1.** Patient counts and percentages with regard to different front-line chemotherapy regimens according to the 8^th^ edition of the TNM Classification.   \|  \| CAP regimen  (n = 45) \| EP regimen  (n = 36) \| TP regimen  (n = 27) \| \| --- \| --- \| --- \| --- \| \| Stages (%)  Stage II  Stage III  Stage IVA  Stage IVB \| 3 (7)  10 (22)  16 (36)  16 (36) \| 1 (3)  6 (17)  10 (28)  19 (53) \| 0 (0)  6 (22)  6 (22)  15 (56) \|   Abbreviations: CAP, cisplatin, doxorubicin, and cyclophosphamide; EP, cisplatin and etoposide; TP, cisplatin and paclitaxel |
| --- | --- | --- | --- | --- | --- | --- | --- | --- |

**Supplementary Table 2.** Progression-free survival and overall survival for stage III/IVA or stage IVB thymoma or thymic carcinoma patients receiving different front-line chemotherapy regimens.

|  | CAP regimen | EP regimen | TP regimen | *p* value |
| --- | --- | --- | --- | --- |
| **Stage III/IVA** | | | | |
| Thymoma (n = 28) | | | | |
| Median PFS | 35.8 months | 28.2 months | 18.0 months | 0.412 |
| 5-year OS | 87.5% | 100% | 100% | 0.765 |
| Thymic carcinoma (n = 30) | | | | |
| Median PFS | 10.4 months | 26.4 months | 12.2 months | 0.676 |
| 5-year OS | 83.3% | 58.3% | 43.8% | 0.599 |
| **Stage** **IVB** | | | | |
| Thymoma (n = 9) | | | | |
| Median PFS | 11.2 months | 2.7 months | NA | 0.228 |
| 5-year OS | 75.0% | 100% | NA | 0.607 |
| Thymic carcinoma (n = 41) | | | | |
| Median PFS | 7.9 months | 7.5 months | 9.5 months | 0.298 |
| 5-year OS | 28.3% | 25.9% | 0 | 0.540 |

Abbreviations: CAP, cisplatin, doxorubicin, and cyclophosphamide; EP, cisplatin and etoposide; NA = not applicable; OS, overall survival; PFS, progression-free survival; TP, cisplatin and paclitaxel

**Supplementary Table 3.** Clinical responses of patients with thymoma or thymic carcinoma after undergoing second-line chemotherapy.

|  | Thymoma (n = 13) | Thymic carcinoma (n = 38) | |
| --- | --- | --- | --- |
| Doxorubicin-based regimens  Response  PR  SD  PD | n = 1 | n = 1  n = 1  n = 4 | |
| Etoposide-based regimens  Response  PR  SD  PD | n = 2  n = 1 | n = 1  n = 2  n = 4 | |
| Taxane-based regimens  Response  PR  SD  PD | n = 1  n = 1 | n = 1  n = 2  n = 2 | |
| Gemcitabine-based regimens  Response  SD  PD |  | n = 1  n = 1 | |
| Fluorouracil -based regimens  Response  PR  SD  PD | n = 1  n = 2 | n = 4  n = 2  n = 2 | |
| Oral cyclophosphamide  Response  SD  PD | n = 3 | n = 6  n = 3 | |
| Pembrolizumab  Response  PD | n = 1 | n = 1 | |
| Abbreviations: PD, progressive disease; PR, partial response; SD, stable disease | | |  |

**Supplementary Table 4.** Characteristics of three patients with stage IVB thymic carcinoma who achieved five-year survival after undergoing front-line treatment.

| No. | Age | First-line C/T | Best response to first-line C/T | Metastatic sites at diagnosis | Second-line C/T | OS (years) |
| --- | --- | --- | --- | --- | --- | --- |
| 1 | 57 | CAP | SD | Lung, lymph nodes | Nil | 5.3 |
| 2 | 62 | CAP | PR | Pleura/pericardium, lymph nodes, bone | EP | 6.8 |
| 3 | 34 | EP | PR | Lung, pleura/pericardium, lymph nodes, bone | Oral cyclophosphamide | 7.1 |
| Abbreviations: C/T, chemotherapy; CAP, cisplatin, doxorubicin, and cyclophosphamide; EP, cisplatin and etoposide; PR, partial response; SD, stable disease; OS, overall survival | | | | | | |
